# Flowers as Dishes With Cultural Flavors: Nutritional Profiles and Taste Characteristics of Four Edible Flower Species in Yunnan, China

**DOI:** 10.1002/fsn3.72253

**Published:** 2026-08-19

**Authors:** Qing Zhang, Yan Li, Wenqi Wang, Miaomiao Wang, Zhuo Cheng, Zhengjiang Xian, Yingzai Zhou, Sarana Rose Sommano, Chunlin Long

**Affiliations:** ^1^ Key Laboratory of Ecology and Environment in Minority Areas (Minzu University of China), National Ethnic Affairs Commission Beijing China; ^2^ College of Ethnology and Sociology Minzu University of China Beijing China; ^3^ School of Chinese Materia Medica Beijing University of Chinese Medicine Beijing China; ^4^ College of Life and Environmental Sciences Minzu University of China Beijing China; ^5^ Longyang Sub‐Bureau of Baoshan Administration of Gaoligongshan National Nature Reserve Yunnan China; ^6^ Tengchong Sub‐Bureau of Baoshan Administration of Gaoligongshan National Nature Reserve Yunnan China; ^7^ Faculty of Agriculture Chiang Mai University Chiang Mai Thailand; ^8^ Institute of National Security Studies Minzu University of China Beijing China

**Keywords:** edible flowers, electronic tongue, human nutrition, local food practices, traditional dietary culture

## Abstract

Flowers are commonly consumed as vegetables and constitute an important component of local diets in many traditional food systems. In regions such as Yunnan, China, edible flowers (EFs) are deeply embedded in biocultural food practices but remain underrepresented in mainstream nutritional and sensory research. This study characterized the nutritional and sensory properties of traditionally consumed EFs of four species (*Musella lasiocarpa*, *Plumeria rubra* , *Stephanotis volubilis*, and *Trevesia palmata*) from Yunnan. Proximate composition, mineral elements, vitamins, and amino acids of four EFs were analyzed, with broccoli used as a nutritional reference due to its well‐recognized status as a nutritionally dense EF, while taste profiles of fresh and blanched samples were assessed using an electronic tongue. All four EFs exhibited distinctive nutrient profiles, including high dietary fiber contents (36.80–57.93 g/100 g dry weight) and comparable or higher levels of several mineral contents than broccoli on a dry‐weight basis. *S. volubilis* contained 10.27 mg/100 g vitamin E, while two species showed particularly high total and essential amino acid contents with favorable amino acid quality indices. Electronic tongue analysis identified CTS (Saltiness) and NMS (Umami) as the dominant taste attributes across species across all four species (*p* < 0.0001), with significantly higher intensities than the other taste indicators both before and after blanching. Blanching altered the intensity of specific taste components (*p* < 0.05, *p* < 0.01, or *p* < 0.001) and induced shifts in the overall taste profiles, as demonstrated by the clear PCA separation between fresh and blanched samples. Nevertheless, each species retained its characteristic sensory identity after processing. These results demonstrate that traditionally used EFs represent nutritionally valuable vegetable resources and provide scientific insights into long‐standing local consumption practices. Despite the bitterness and other taste characteristics that may limit palatability, these flowers remain popular food resources in Yunnan, suggesting that factors such as cultural context may contribute to their continued use. Integrating ethnobotanical knowledge with nutritional and sensory analyses provides a robust framework for understanding EFs and supports their sustainable use and food cultural heritage within local diet systems.

## Graphical abstract

This graphical abstract highlights the nutritional profiles and taste characteristics of four edible flower species, *Musella lasiocarpa* (Franch.) C. Y. Wu ex H. W. Li, *Plumeria rubra* L. “Acutifolia,” *Stephanotis volubilis* (L.f.) S.Reuss, Liede & Meve (syn. *Dregea volubilis* (L. f.) Benth. ex Hook. f.) and *Trevesia palmata* (Roxb.) Vis., which are traditionally consumed in Yunnan, China. It illustrates their deep‐rooted cultural culinary uses, reveals their distinct nutritional advantages, including dietary fiber, minerals, vitamin E, and high‐quality amino acid profiles, and employs E‐tongue sensory analysis to demonstrate how blanching modifies taste intensity while preserving species‐specific flavors. The findings position these flowers as valuable biocultural food resources that nourish both people and culture.

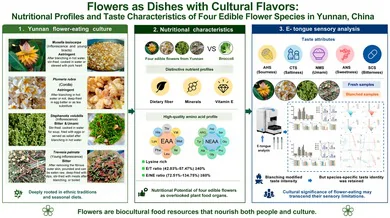

## 1 Introduction

Flowers have long been widely valued for their ornamental qualities, yet their role as food remains less studied in both culinary practice and scientific research (Purohit et al. 2021). Both traditional knowledge and contemporary research demonstrate that flowers have valuable edible properties and constitute distinctive culinary ingredients (Liu et al. 2023). In regions such as the Mediterranean, Mexico, and many Asian countries, the consumption of flowers is embedded within rich local knowledge systems and marked cultural practices (Khomdram et al. 2019; Motti et al. 2022; Rodrigues and Spence 2023; Samkaria and Kumari 2025). For example, edible flowers (EFs) play a central role in sacred Mexican rituals and serve as festival foods among ethnic groups in Xishuangbanna, Yunnan (e.g., the Dai and Yao peoples) (Mulík and Ozuna 2020; Zhang, Cheng, et al. 2023).

**FIGURE 1:**
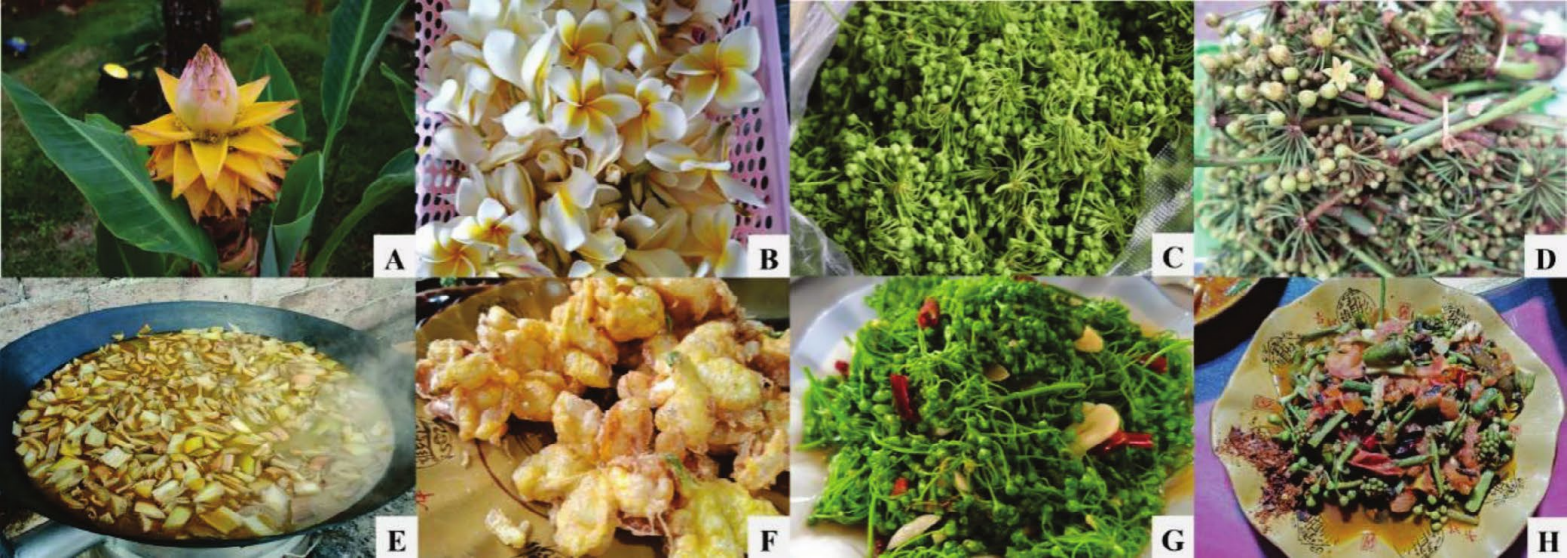
Four EFs and dishes. (A) *Musella lasiocarpa*; (B) *Plumeria rubra* “Acutifolia”; (C) *Stephanotis volubilis*; (D) *Trevesia palmata*; (E) stewed chopped inflorescences and young bracts of *M. lasiocarpa* with mutton; (F) *P. rubra* “Acutifolia” corolla coated in beaten egg and deep‐fried; (G) stir‐fried inflorescences of *S. volubilis* ; (H) stir‐fried young inflorescences of *T. palmata* with tomatoes.

Yunnan, the richest biocultural diverse province in China, hosts a rich and widespread tradition of consuming flowers, forming a unique flower‐eating culture (Wen et al. 2025; Zhang, Cheng, et al. 2023; Zhang, Zhang, et al. 2025). A common saying among local people, “fresh flowers are treated as vegetables,” reflects the long‐standing culinary traditional culture of using flowers as everyday food. A survey of EFs consumed as vegetables conducted across markets and restaurants in 102 counties and cities of Yunnan, involving 679 interviewees, documented 140 species belonging to 108 genera and 52 families (Yang et al. 2017). Local understandings of EFs, especially perceptions of their dietary benefits and flavor profiles, varies across species (Liu and Long 2001; Yang et al. 2017; Zhang, Zhang, et al. 2025). *Musella lasiocarpa* (Franch.) C. Y. Wu ex H. W. Li, *Plumeria rubra* L. ‘Acutifolia’, *Stephanotis volubilis* (L.f.) S.Reuss, Liede & Meve (syn. *Dregea volubilis* (L. f.) Benth. ex Hook. f.) and *Trevesia palmata* (Roxb.) Vis. (Figure 1) are widely consumed in Yunnan. The ethnobotanical study on EFs in Xishuangbanna, Yunnan, shows relative high frequency of citation of these EFs of four species, indicating the important roles they play in local flower‐eating cuisine (Zhang, Cheng, et al. 2023).

Both *Musella lasiocarpa* (Musaceae) and *Plumeria rubra* (Apocynaceae) are popular ornamental and religious plants in East and Southeast Asia, designated as two of the “Five Trees and Six Flowers” sacred in Buddhism (Xu et al. 2023; Bihani 2021; Yang et al. 2023; Albuquerque‐Lima et al. 2025). They are widely cultivated in Buddhist gardens across southern China (particularly Yunnan) and Southeast Asia (Wang et al. 2014). *M. lasiocarpa*, a perennial herbaceous plant endemic to southwestern China (Albuquerque‐Lima et al. 2025), has long been utilized by local people for medicine, fodder, food, ornamental and weaving materials (Liu et al. 2003; Long et al. 2008). Among its parts, the inflorescence as forage was found to have higher nutritional quality than the stem, leaf blade, and petiole (Li et al. 2023), and its consumption is regarded as an important component of flower‐eating culture in Yunnan (Li, Zhang, et al. 2025). However, comprehensive information on the nutritional composition of *M. lasiocarpa* inflorescence as a human food source remains limited. *Plumeria rubra* is a deciduous small tree native to Mexico, Central America, Colombia and Venezuela, now widely cultivated across tropical and subtropical Asia for ornamental, medicinal and cultural purposes (POWO 2026). Its flowers, leaves, bark, roots, and fruits of *P. rubra* are widely used in ethnomedicine for the treatment of 37 different diseases across various regions of the world (Bihani 2021). In China, the medicinal use of *P. rubra* flowers was first documented in *Lingnan Caiyao Lu* (Medicinal Records of Lingnan published in 1932), for treating damp‐heat dysentery, tenesmus, and moistening the lungs. It remains widely used today as a major ingredient in Guangdong herbal tea (*liang cha*) (Deng et al. 2014). Nevertheless, its potential as a food resource has received far less attention than its medicinal uses.

*Stephanotis volubilis* (Apocynaceae) and *Trevesia palmata* (Araliaceae) are native to China and primarily grow in the wet tropical biomes (POWO 2026). Both species are medicinal food plants commonly consumed in South China, Thailand, Myanmar, Lao PDR and India (Cao et al. 2020; Zhang et al. 2020; Sommano et al. 2025; Lalmuanpuii et al. 2024; Lamxay et al. 2011). For example, in central Myanmar markets, *S. volubilis* is the most frequently sold wild vegetable, with its blanched flowers commonly prepared as salads (Zhang et al. 2020). The flower buds of *T. palmata* are eaten as a wild vegetable in Northeast India (Lalmuanpuii et al. 2024). Studies found that both species contain relatively high levels of total amino acids, with flower buds of *T. palmata* showing a higher protein content and *S. volubilis* inflorescences being particularly rich in phenolic compounds (Sommano et al. 2025). Das et al. (2017) reported that *S. volubilis* flower extracts are rich in phenolics and flavonoids and display significant antioxidant activity and inhibitory effects on *α*‐amylase and *α*‐glucosidase, suggesting nutraceutical and antidiabetic potential. As bitter‐tasted medicinal food plants, *S. volubilis* and *T. palmata* may possess valuable nutritional and bioactive properties, yet research on these aspects remains scarce (Leonti et al. 2024).

Many studies have verified the dietary and health potential of EFs through analyses of their nutritional composition, bioactive compounds, and antioxidant activity. Floral parts such as pollen, nectar, and corollas are rich in proteins, amino acids, carbohydrates, lipids, vitamins, minerals, antioxidants, and so forth (Mlcek and Rop 2011). Rop et al. (2012) reported the mineral composition of 12 EFs exceeded that of most fruits and vegetables. Fernandes et al. (2017) summarized that carbohydrates are likely the most abundant nutrient group in 29 common EFs. Rachkeeree et al. (2018) analyzed the nutritional and phytochemical properties of 8 Zingiberaceous EFs widely consumed in Thailand, some of which were rich in dietary fiber, macroelements such as potassium and calcium, as well as antioxidants. Similarly, four wild EFs were reported to contain high levels of dietary fiber, calcium, iron, and carbohydrates (Suksathan et al. 2021). Collectively, these studies provide scientific evidence supporting the role of EFs as potential sources of essential nutrients. However, most of these investigations were not designed from the perspective of traditional food knowledge, nor did they relate analytical results to local dietary practices and cultural contexts.

Sensory characteristics are a key factor influencing the collection, processing, and acceptance of EFs, with species exhibiting distinct sensory profiles such as mild sweetness, bitterish, or slight sour (Mlcek and Rop 2011). Currently, the sensory evaluation of EFs primarily relies on artificial sensory descriptive analysis (Demasi et al. 2021; Mateo‐Nakamura et al. 2026), which can provide detailed sensory descriptions but suffers from subjectivity, time consumption, and environmental interference (Köster 2009; Varela and Ares 2012). As a relatively novel taste detection technology, the electronic tongue (e‐tongue) offers objective, efficient, and highly reproducible taste detection using lipid membrane sensors that quantify basic tastes (sourness, sweetness, bitterness, saltiness, umami) (Deisingh et al. 2004; Vanaraj et al. 2025; Ciosek and Wróblewski 2007). Although the e‐tongue has been applied in the field of food sensory science (Adeyeye and Adeyeye 2025; Escuder‐Gilabert and Peris 2010), its use in studying the sensory characteristics of EFs has not yet been reported.

To date, nutritional study on Yunnan's EFs is still limited, with existing studies covering only a small number of species. However, the available evidence consistently indicates that these species possess high nutritional value and represent promising food resources. Yin et al. (2010) analyzed and evaluated the nutritional composition of 11 commonly and traditionally consumed wild EFs from Yunnan using the Chinese national standard methods. The results showed that although the nutrient contents varied among species, most of the flowers were rich in protein and minerals, characterized by high potassium and low sodium levels, and contained abundant dietary fiber while being low in calories and fat. Zhang, Cao, et al. (2023) conducted a comprehensive analysis on 34 EFs from Yunnan. Although these species are not traditionally consumed as food in the region, the results revealed their high nutritional value and strong antioxidant capacity. In addition, the folk applications and phytochemical research of 10 Yunnan EFs were reviewed, yet lacked perspectives from nutrition and food science (Wen et al. 2025). Therefore, systematic nutritional and sensory evaluations of culturally important, traditionally consumed EFs are necessary.

Although flowers have a long history of consumption in certain regions and among specific linguistic groups, they are also emerging as novel food resources in many other places (Fernández‐Pintor et al. 2025). Given that different EFs exhibit distinct nutritional compositions, it is essential to develop species‐specific knowledge and evaluation for each of them (Fernandes et al. 2017). However, nutritional studies of EFs in Yunnan have focused on a limited number of species, and comprehensive data for many culturally important flowers consumed in Yunnan are still lacking. Most existing studies emphasize nutritional composition, bioactive compounds, or antioxidant activity, whereas sensory characteristics—an important factor influencing food acceptance and use—have received much less attention. In addition, few studies have integrated nutritional and sensory evaluations within the context of traditional food knowledge and actual consumption practices. Addressing these gaps is essential for a more complete understanding of EFs as foods rather than merely as sources of functional compounds. The present study focuses on four EF species that are traditionally consumed as vegetables in Yunnan, selected based on ethnobotanical evidence and relatively high citation frequencies, but for which nutritional information remains limited (Zhang, Cheng, et al. 2023). All four species have been consumed locally for generations without reported adverse effects, confirming their safety as edible flowers. Through nutritional and e‐tongue tests, this study aims to explore and reveal the nutrient profiles and sensory characteristics of four EFs, providing further evidence for flowers as consumable plant organs, while validating long‐standing community practices, supporting food cultural heritage and highlighting their role in sustainable diets.

## 2 Materials and Methods

### 2.1 Plant Collection and Identification

The plant samples were obtained from local markets or cultivation gardens, which are the primary sources for obtaining edible flowers in the area. Ethnobotanical data, plant characteristics, traditional consuming culture were recorded (Table 1). After confirmation with local informants, these samples were collected just before they were to be harvested and consumed by the local people, ensuring alignment with local practices. Prof. Chunlin Long identified the plant species, and the voucher specimens were deposited in the herbarium at Minzu University of China in Beijing.

**TABLE 1:** Identified edible flowers consumed by people in Yunnan and samples information.

Scientific name	*Musella lasiocarpa* (Franch.) C. Y. Wu ex H. W. Li	*Plumeria rubra* L. “Acutifolia”	*Stephanotis volubilis* (L.f.) S.Reuss, Liede & Meve	*Trevesia palmata* (Roxb.) Vis.
Family name	Musaceae	Apocynaceae	Apocynaceae	Araliaceae
Chinese name	地涌金莲	鸡蛋花	南山藤/苦藤花	刺通草
Vernacular name among linguistic groups in Yunnan	di lian hua (HAN), nga dou (YI)	muo ke zhang ba (DA), miao zhi zi (HAN)	a ka ba a ba/teng ke ou ni (HN), ye ya/gong gai fang (YA), lao mei hong/log pa ong (DA), pa ke lou ar bu. (JN), ku teng (HAN)	you dang/da wan/dang bie (DA), bie da (HN), ben gong (YI), tai (JN), ku liang bao (HAN)
Consumed parts of the Floral Organ	Inflorescence and young bracts	Corolla	Inflorescence	Young inflorescence
Methods of Preparation for Food	After blanching in hot water, stir‐fried, cooked in water or stewed with pork heart	After blanching in hot water or not, deep‐fried in egg batter or as tea substitute	Stir‐fried, cooked in water for soup, fried with eggs or served as salad after blanching in hot water	After removing the fibrous outer skin, pounded and can be eaten raw, deep‐fried with dips, stir‐fried with meats after blanching, or boiled
Taste of the flowers	Astringent	Astringent	Bitter and umami	Bitter
Sampling sites for nutritional test	From Donghua, Chuxiong Jan. 2023	From Menglun, Xishuangbanna Nov. 2023	From Yuanjiang, Yuxi Sept. 2023	From Menglun, Xishuangbanna Mar. 2023
Sampling sites for E‐tongue test	From Lujiang, Baoshan Mar. 2025	From Menglun, Xishuangbanna Apr. 2025	From Menglun, Xishuangbanna Apr. 2025	From Lujiang, Baoshan Mar. 2025
Voucher Number	BN179	BN163	BN037	BN120

Abbreviations: DA, Dai ethnic group; HAN, Han people (the majority in China); HN, Hani ethnic group; JN, Jinuo ethnic group; YA, Yao ethnic group; YI, Yi ethnic group.

### 2.2 Nutritional Analysis and Evaluation

#### 2.2.1 Proximate Analysis

We conducted proximate analysis to determine the nutritional composition of four EFs. The analyzed parameters included moisture, ash, fat, total dietary fiber, crude protein, carbohydrate and energy value. All measurements were strictly performed in accordance with the National Standards of the People's Republic of China (GB standards) for food composition determination (National Health and Family Planning Commission of PRC 2014, 2016a, 2016b, 2016c, 2016d, 2016e, 2016f, 2016g, 2016h, 2016i, 2016j, 2016k, 2017).

Following the direct drying method of the GB 5009.3‐2016, the moisture content was determined with the drying oven set at 103°C. The ash content was determined in strict accordance with the method for “Foods with High Phosphorus Content and Other Foods” as specified in the GB 5009.4‐2016. The sample was incinerated at 550°C for 30 min. Total fat content was determined by the solvent extraction method according to the GB 5009.6‐2016. Protein content was determined by Kjeldahl nitrogen analysis according to the GB 5009.5‐2016. Total dietary fiber (TDF) was determined according to the GB 5009.88‐2014. Sample preparation followed the specific procedures for “samples with fat content < 10%” and “moisture content < 10%,” followed by enzymatic hydrolysis and subsequent analysis as stipulated in the standard. Total carbohydrates were calculated by subtracting the total percentage of other components (saccharides, starch, and dietary fiber) from 100 according to the GB/Z 21922‐2008 (Ministry of Health of PRC & Standardization Administration of PRC 2008). Carbohydrate content was calculated according to the formula:

(1) Carbohydrates=100−Protein−Fat−Moisture−Ash−DietaryFiber

Energy content was calculated according to the formula:

(2) $$\begin{array}{c} \text{Energy}=\text{Protein}\times 17+\text{Carbohydrates}\times 17+\mathrm{Fat}\times 37+\text{Dietary Fiber}\times 8 \end{array}$$

#### 2.2.2 Mineral Composition

The mineral composition of 10 elements: copper (Cu), zinc (Zn), iron (Fe), calcium (Ca), magnesium (Mg), potassium (K), sodium (Na), manganese (Mn), phosphorus (P), and selenium (Se) was determined in the four EF samples. The analyses of Cu, Zn, Fe, Ca, Mg, K, Na, Mn, and P were conducted using Inductively Coupled Plasma Optical Emission Spectrometry (ICP‐OES; the second method of GB 5009.268‐2016). Due to its typically lower concentration and higher detection sensitivity requirements, Se was analyzed using Inductively Coupled Plasma Mass Spectrometry (ICP‐MS; the first method of GB 5009.268‐2016).

#### 2.2.3 Vitamin Composition

The contents of specific vitamins were determined using chromatographic and microbiological methods with reference to the corresponding Chinese National Standards. Vitamin A and vitamin E were analyzed by reversed‐phase high‐performance liquid chromatography (RP‐HPLC) according to GB 5009.82‐2016 (Method 1). Vitamin D was determined by high‐performance liquid chromatography (HPLC) following Method 4 of the same standard. Vitamins B_1_, B_2_, and C were quantified using HPLC in accordance with the first method of GB 5009.84‐2016, GB 5009.85‐2016, and GB 5009.86‐2016, respectively. Vitamin K_1_ was analyzed by HPLC with fluorescence detection (HPLC‐FLD) according to GB 5009.158‐2016 (Method 1). Vitamin B_6_ was determined using a microbiological assay as specified in GB 5009.154‐2016 (Method 2).

#### 2.2.4 Amino Acid Composition

The amino acid composition was analyzed following acid hydrolysis using an amino acid analyzer in accordance with the Chinese National Standard GB 5009.124‐2016. A total of 16 amino acids were quantified, including aspartic acid, threonine, serine, glutamic acid, proline, glycine, alanine, valine, methionine, isoleucine, leucine, tyrosine, phenylalanine, lysine, histidine, and arginine. Based on the measured contents, the total amounts and proportions of essential amino acids (EAA) and non‐essential amino acids (NEAA) were calculated for each of the four EF species.

#### 2.2.5 Data Comparison and Analysis

For comparative evaluation, broccoli ( *Brassica oleracea var. italica* Plenck) was selected as the primary reference vegetable because it is one of the most widely consumed EFs as a vegetable worldwide. Furthermore, it is well recognized as a nutritious and health‐promoting food, particularly as a source of macronutrients (Andrés et al. 2025; Zhang, Jia, et al. 2025). The proximate composition, vitamin contents, and mineral elements of the four studied EFs were compared with corresponding values for broccoli reported in the China Food Composition Tables (Standard Edition) (National Institute for Nutrition and Health Chinese Center for Disease Control and Prevention 2018).

Because the EF samples were analyzed on a dry‐weight (DW) basis, whereas the broccoli reference data were originally reported on a fresh‐weight (FW) basis, broccoli values were converted to DW equivalents prior to comparison using standard moisture‐based conversion equations (% DW = % FW/[1 – *M*], where *M* = moisture content/100) (AFSI Crop Composition Database 2024). Moisture contents of 91.6 g/100 g FW for broccoli from the *China Food Composition Tables*. These comparisons were intended to contextualize the nutritional characteristics of traditional EFs relative to a commonly consumed and nutritionally well‐characterized vegetable while ensuring consistency in the basis of nutrient expression.

All experimental measurements were repeated at least three times and results are expressed as mean ± standard deviation (SD) calculated using SPSS 26.0.

### 2.3 Electronic Tongue Analysis

Blanching serves as a conventional and, in most cases, indispensable pretreatment for all four EFs in local culinary practices, employed both to reduce undesirable bitterness and eliminate potential toxicity, thereby preparing the flowers for subsequent cooking. To clarify the taste profile characteristics of the four EFs in their fresh state and after blanching, an electronic tongue was employed to conduct taste component analysis on the corresponding samples. Five core taste indices, sourness, saltiness, umami, sweetness and bitterness, were determined. It was hypothesized that fresh samples represent the most authentic taste characteristics of the EFs, while blanching, a critical pretreatment step for local consumption, would alter their inherent flavor profiles.

#### 2.3.1 Samples Preparation

The samples of four EFs were processed and e‐tongue testing was performed at the School of Chinese Materia Medica, Beijing University of Chinese Medicine. To reflect local consumption practices, where these flowers are typically eaten either fresh or blanched, we prepared each flower type using both methods. For fresh samples, only the floral organs recorded as the consumed portions in the ethnobotanical survey were used. Other plant parts were removed and excluded from analysis. The consumed portions were washed, air‐dried, and approximately 50 g of edible material was weighed out. The material was ground in a mortar with 100 mL of deionized water to extract the juice, and the resulting homogenate was transferred to 50‐mL centrifuge tubes and centrifuged at 10,000 rpm for 15 min. The supernatant was collected into a new 50‐mL tube and used as the fresh sample solution. For blanched samples, a separate set of specimens was prepared in the same manner, with 50 g of edible portions weighed out. The samples were blanched in 400 mL of boiling deionized water and simmered for 3–5 min depending on flower type. After coarse filtration, the blanched material was ground in a mortar with 100 mL of the filtrate, followed by centrifugation and supernatant collection using the same procedures as for the fresh samples, yielding the blanched sample solutions. All prepared solutions were stored at −80°C until e‐tongue analysis.

#### 2.3.2 Electronic Tongue Analysis

The ASTREE electronic tongue system (Alpha M.O.S., Toulouse, France) used in this study is equipped with five taste sensors and two reference sensors for analyzing the flavor profile of EFs. The five taste sensors are AHS (Sourness), CTS (Saltiness), NMS (Umami), ANS (Sweetness), and SCS (Bitterness), along with two general‐purpose sensors, PKS and CPS. The sensor measurement process mainly consists of four steps: pre‐equilibration, calibration, data acquisition, and data processing. First, pre‐equilibration is performed by conditioning the sensors in deionized water and 0.01 mol L^−1^ HCl solution to ensure stable level with minimal noise drift. This is followed by calibration, during which each sensor is adjusted to preset target values while keeping the error within an acceptable range. During the data acquisition stage, the sensors are immersed in the test solution for 120 s, and the measurement is repeated nine times. After each measurement, the sensors are rinsed twice with fresh deionized water (30 s each rinse, six rinses in total) to prevent cross‐contamination. Finally, in the data processing stage, data from the first four measurements are used for sensor training, while data from the fifth measurement (100–120 s) are extracted for subsequent analyses, including principal component analysis (PCA) and radar chart analysis.

#### 2.3.3 Statistical Analysis

Each sample was tested in five replicates, with data presented as mean ± standard deviation (mean ± SD). Normality was confirmed using the Shapiro–Wilk test (*p* > 0.05) by GraphPad Prism 9.5 (GraphPad Software, Boston, MA, USA). Two‐way analysis of variance (two‐way ANOVA) assessed the effects of flower species, taste type, and their interaction on taste scores; significant differences (*p* < 0.05) were further analyzed with Tukey's honest significant difference (HSD) test for pairwise comparisons. Intra‐group and inter‐group analyses were performed on the fresh and blanched samples of the four EFs, respectively. Changes in taste indices before and after blanching were compared for each flower species. A paired *t*‐test was used to analyze the changes. Radar plots and PCA were used to elucidate the effects of different processing methods on their taste profiles. Radar charts were constructed in Origin 2018 (OriginLab Corporation, Northampton, MA, USA). PCA was performed on the online platform https://www.bioinformatics.com.cn (accessed 10 Dec 2024) to visualize sample clustering patterns. Statistical significance was set at *p* < 0.05.

## 3 Results

### 3.1 Nutrient Compositions

#### 3.1.1 Proximate Compositions

The proximate compositions of four EFs and broccoli ( *B. oleracea var. italica* ) were shown in Table 2. Energy values for the flowers ranged from 937 to 1080 kJ/100 g, compared with 1321 kJ/100 g in broccoli. *T. palmata* (24.67/100 g) and *S. volubilis* (20.40 g/100 g) showed particularly high protein concentrations, while *M. lasiocarpa* and *P. rubra* contained 9.07 and 8.27/100 g, respectively; all were lower than broccoli (41.67 g/100 g). Fat contents were relatively low in all four flowers, ranging from 0.90 g/100 g in *T. palmata* to 5.60 g/100 g in *P. rubra* , and were below the broccoli value (7.14 g/100 g).

**TABLE 2:** Proximate composition, vitamin, and mineral elements of 4 edible flowers from Yunnan and *B. oleracea var. italica* . Results are presented as mean ± standard deviation (values per 100 g dry‐weight of samples basis).

Items	Unit	*M. lasiocarpa*	*P. rubra*	*S. volubilis*	*T. palmata*	*B. oleracea var. italica* ^a^
Energy	kJ	937	1080	1059	1042	1321
Protein	g	9.07 ± 0.03	8.27 ± 0.10	20.40 ± 0.01	24.67 ± 0.06	41.67
Fat	g	1.43 ± 0.06	5.60 ± 0.00	1.20 ± 0.00	0.90 ± 0.00	7.14
Dietary fiber	g	57.93 ± 0.85	48.47 ± 0.74	37.37 ± 0.91	36.80 ± 0.50	19.05
Carbohydrate	g	15.7	20.2	21.7	17.3	44.05
Moisture	g	8.69 ± 0.08	10.97 ± 0.06	9.71 ± 0.10	8.17 ± 0.06	
Ash	g	7.20 ± 0.10	6.37 ± 0.06	9.63 ± 0.06	12.07 ± 0.06	7.14
Vitamins						
Vitamin A	μg	< 30.0	< 30.0	< 30.0	< 30.0	154.76
Vitamin B_1_	mg	< 0.1	< 0.1	0.13 ± 0.00	< 0.1	0.71
Vitamin B_2_	mg	< 0.05	0.56 ± 0.00	0.32 ± 0.01	0.42 ± 0.01	0.95
Vitamin B_6_	mg	0.29 ± 0.01	0.10 ± 0.00	0.37 ± 0.01	0.32 ± 0.01	
Vitamin C	mg	< 2.0	8.29 ± 0.04	88.53 ± 0.55	< 2.0	666.67
Vitamin D	μg	< 2.0	< 2.0	< 2.0	< 2.0	
Vitamin E	mg	4.43 ± 0.02	3.41 ± 0.10	10.27 ± 0.23	4.52 ± 0.01	9.05
Vitamin K_1_	mg	0.02 ± 0.00	0.01 ± 0.00	0.18 ± 0.00	0.33 ± 0.01	
Mineral content						
Ca	mg	700.33 ± 9.61	638.33 ± 1.03	956.67 ± 23.54	149.00 ± 1.00	595.24
P	mg	295.00 ± 7.55	172.67 ± 3.51	474.33 ± 8.08	453.33 ± 23.29	726.19
Mg	mg	219.00 ± 4.58	118.33 ± 2.51	328.00 ± 5.57	423.33 ± 18.01	261.90
K	mg	2693.33 ± 45.09	2200.00 ± 36.06	3923.33 ± 85.05	3953.33 ± 128.58	2130.95
Na	mg	4.87 ± 0.11	4.54 ± 0.11	5.52 ± 0.28	5.96 ± 0.35	555.95
Zn	mg	2.44 ± 0.06	1.54 ± 0.04	3.03 ± 0.04	7.06 ± 0.08	5.48
Fe	mg	8.67 ± 0.10	6.65 ± 0.14	7.92 ± 0.13	7.19 ± 0.21	10.71
Mn	mg	5.86 ± 0.10	1.04 ± 0.02	5.10 ± 0.13	9.61 ± 0.42	1.90
Cu	mg	0.36 ± 0.02	0.85 ± 0.03	1.04 ± 0.02	0.80 ± 0.02	0.36
Se	μg	< 3.0	< 3.0	3.72 ± 0.22	< 3.0	5.12

^a^ From Chinese Food Composition Table.

Dietary fiber was the clearest nutritional advantage of the four flowers. Values ranged from 36.80/100 g in *T. palmata* to 57.93 g/100 g in *M. lasiocarpa*, far exceeding broccoli (19.05 g/100 g). Carbohydrate contents in the flowers were lower than in broccoli (44.05 g/100 g), varying from 15.7 g/100 g in *M. lasiocarpa* to 21.7 g/100 g in *S. volubilis* . Ash content, an index of total mineral matter, ranged from 6.37 to 12.07 g/100 g. Compared with broccoli (7.14 g/100 g), *S. volubilis* and *T. palmata* showed higher values; *M. lasiocarpa* was similar and *P. rubra* was slightly lower, indicating that the four EFs generally contain substantial mineral loads.

#### 3.1.2 Mineral Elements

In the study, a total of 10 minerals, including calcium (Ca), phosphorus (P), magnesium (Mg), potassium (K), sodium (Na), zinc (Zn), iron (Fe), manganese (Mn), copper (Cu) and selenium (Se) were investigated. The four EFs contained an array of essential minerals and several vitamins at concentrations that in some cases exceed those of broccoli (Table 2). The minerals K was especially abundant in all flower samples, ranging from 2200.00 mg/100 g in *P. rubra* to 3953.33 mg/100 g in *T. palmata* and 3923.33 mg/100 g in *S. volubilis* , and all values exceeded broccoli (2130.95 mg/100 g). Ca concentrations were high in *S. volubilis* (956.67.00 mg/100 g) and *M. lasiocarpa* (703.33 mg/100 g), while *P. rubra* also contained substantial Ca (638.33 mg/100 g). They exceed the broccoli Ca level (595.24 mg/100 g). Magnesium (Mg) contents were lower than broccoli in *M. lasiocarpa* and *P. rubra* , but higher in *S. volubilis* and *T. palmata*, reaching 328.00 and 423.33 mg/100 g, respectively. Phosphorus (P) was lower in all four flowers than in broccoli, although *S. volubilis* and *T. palmata* still showed relatively high values. Sodium (Na) was very low in all four flowers (4.54–5.96 mg/100 g) and notably lower than in broccoli (555.95 mg/100 g), indicating a favorable low‐sodium profile.

Trace elements were present at appreciable levels. Zinc (Zn) was lower than broccoli in *M. lasiocarpa*, *P. rubra* , and *S. volubilis* , but higher in *T. palmata* (7.06 mg/100 g). Iron (Fe) was detected in all species but was lower than broccoli in each case, ranging from 6.65 to 8.67 mg/100 g. Manganese (Mn) was higher than broccoli in *M. lasiocarpa*, *S. volubilis* , and *T. palmata*, with the highest level observed in *T. palmata* (9.61 mg/100 g). Copper (Cu) was present at elevated concentrations in the flowers (0.36–1.04 mg/100 g) versus broccoli (0.36 mg/100 g). Selenium (Se) was below detection in most flower samples (< 3 μg/100 g) but detected at 3.72 μg/100 g in *S. volubilis* which was lower than 5.12 μg/100 g in broccoli.

#### 3.1.3 Vitamins

Vitamin analysis showed a consistent presence of vitamin B_6_, vitamin E and vitamin K_1_ in all flower samples, whereas vitamins A and D were all below detection limits (Table 2). *S. volubilis* contained particularly high vitamin C (88.53 mg/100 g), whereas *P. rubra* had moderate vitamin C (8.29 mg/100 g) and the other species were below the detection threshold for vitamin C. Vitamin E concentrations were notable in *S. volubilis* (10.27 mg/100 g) compared with broccoli (9.05 mg/100 g). Vitamin K_1_ was abundant in *T. palmata* (0.33 mg/100 g) and *S. volubilis* (0.18 mg/100 g), while *M. lasiocarpa* and *P. rubra* contained lower but detectable K1 amounts (0.02 and 0.01 mg/100 g, respectively).

B‐group vitamins showed variation among the four EFs and generally lower than those detected in broccoli. Vitamin B_1_ was detected only in *S. volubilis* (0.13 mg/100 g), while it was below the detection limit in the other three species. Vitamin B_2_ was present in *S. volubilis* (0.32 mg/100 g), *P. rubra* (0.56 mg/100 g), and *T. palmata* (0.42 mg/100 g), but was below the detection limit in *M. lasiocarpa*; all three detectable values were lower than broccoli (0.95 mg/100 g). Vitamin B_6_ was detected in all four EFs, with concentrations of 0.29 mg/100 g in *M. lasiocarpa*, 0.37 mg/100 g in *S. volubilis* , and 0.32 mg/100 g in *T. palmata*. Overall, *S. volubilis* was the only species whose vitamin E content exceeded that of broccoli, while most other detected vitamins occurred at lower concentrations than those reported for the reference vegetable.

#### 3.1.4 Amino Acid Composition

The amino acid compositions of the four EFs are presented in Table 3. Sixteen amino acids were detected in the four EFs, encompassing the total content of eight essential amino acids (EAA) and eight non‐essential amino acids (NEAA). Total amino acid (TAA) contents of *S. volubilis* (17.77 g/100 g) and *T. palmata* (16.53 g/100 g) were substantially higher than those of *M. lasiocarpa* (5.68 g/100 g) and *P. rubra* (5.90 g/100 g). *S. volubilis* and *T. palmata* exhibited markedly higher EAA levels than *M. lasiocarpa* and *P. rubra* . Among individual EAAs, lysine was particularly abundant in *S. volubilis* (5.87 g/100 g) and *T. palmata* (5.49 g/100 g). Other EAAs, including leucine, isoleucine, valine, phenylalanine and histidine, showed the same general trend, with higher levels in *S. volubilis* and *T. palmata* than in the other two species. Methionine was present at low levels in all species. Among the NEAAs, aspartic acid and glutamic acid were the most abundant in *S. volubilis* , while *T. palmata* showed relatively high levels of glutamic acid and arginine.

**TABLE 3:** Amino acids of 4 edible flowers from Yunnan.

Amino acid type (g/100 g)	*M. lasiocarpa*	*P. rubra*	*S. volubilis*	*T. palmata*
Threonine	0.21 ± 0.01	0.33 ± 0.01	0.40 ± 0.01	0.57 ± 0.02
Valine	0.28 ± 0.00	0.37 ± 0.01	0.51 ± 0.01	0.60 ± 0.01
Methionine	0.01 ± 0.00	0.01 ± 0.00	< 0.01	0.02 ± 0.00
Isoleucine	0.19 ± 0.00	0.31 ± 0.01	0.38 ± 0.01	0.48 ± 0.01
Leucine	0.35 ± 0.01	0.57 ± 0.01	0.70 ± 0.00	0.83 ± 0.01
Phenylalanine	0.22 ± 0.00	0.43 ± 0.01	0.55 ± 0.00	0.63 ± 0.02
Lysine	1.34 ± 0.02	0.30 ± 0.01	5.87 ± 0.10	5.49 ± 0.08
Histidine	0.28 ± 0.00	0.16 ± 0.00	0.83 ± 0.00	0.87 ± 0.02
EAA	2.89 ± 0.01	2.48 ± 0.04	9.24 ± 0.09	9.50 ± 0.12
Aspartic acid	0.56 ± 0.00	0.68 ± 0.01	4.05 ± 0.09	1.67 ± 0.02
Serine	0.27 ± 0.01	0.36 ± 0.01	0.48 ± 0.01	0.58 ± 0.02
Glutamate acid	0.91 ± 0.02	0.73 ± 0.01	2.02 ± 0.03	1.16 ± 0.02
Proline	0.16 ± 0.00	0.34 ± 0.01	0.31 ± 0.00	0.77 ± 0.02
Glycine	0.25 ± 0.01	0.37 ± 0.01	0.47 ± 0.01	0.75 ± 0.01
Alanine	0.32 ± 0.01	0.39 ± 0.01	0.49 ± 0.01	0.63 ± 0.01
Tyrosine	0.08 ± 0.00	0.23 ± 0.00	0.23 ± 0.00	0.31 ± 0.01
Arginine	0.25 ± 0.00	0.32 ± 0.01	0.47 ± 0.01	1.17 ± 0.03
NEAA	2.80 ± 0.03	3.42 ± 0.02	8.53 ± 0.07	7.05 ± 0.07
TAA	5.68 ± 0.03	5.90 ± 0.07	17.77 ± 0.15	16.53 ± 0.21
E/T	50.88%	42.03%	52.00%	57.47%
NE/T	49.30%	57.97%	48.00%	42.65%
E/NE	103.21%	72.51%	108.32%	134.75%

The percentage of EAAs in TAAs (E/T) ranged from 42.03% in *P. rubra* to 57.47% in *T. palmata*. According to the WHO/FAO ideal amino acid pattern, an E/T ratio of approximately 40% and an EAA/NEAA ratio (E/NE) of ≥ 60% are considered favorable for human nutrition (Zhao et al. 2023). All four EFs exceeded these criteria, indicating relatively favorable EAA proportions across all four species.

### 3.2 Taste Characteristics of Four EFs

#### 3.2.1 Intra‐Group and Inter‐Group Differences in Taste Profiles of Four EFs in Fresh Status

**FIGURE 2:**
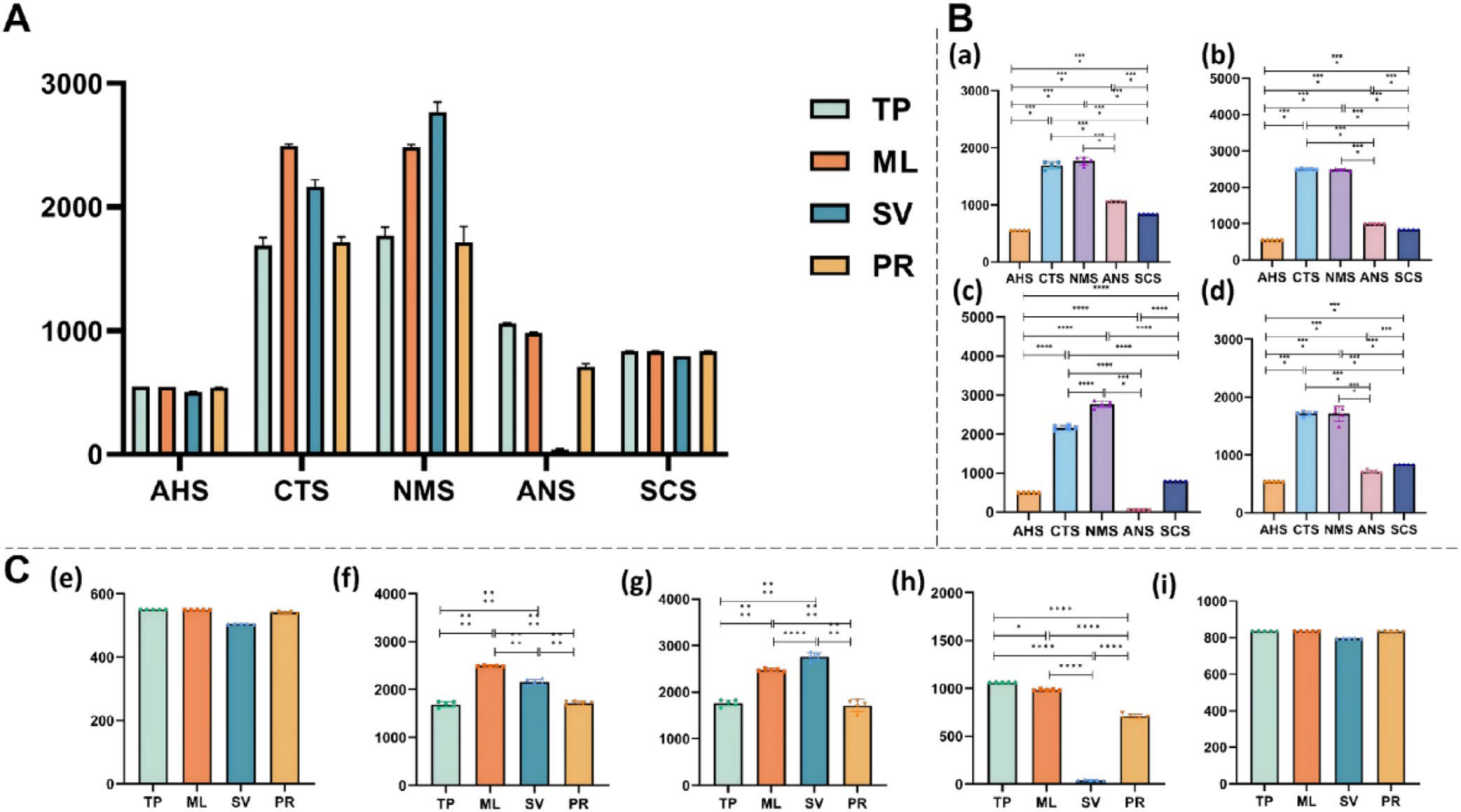
Intra‐group and inter‐group comparisons of four fresh EFs. (A) Overview. (B) Intra‐group comparison of taste attributes: (a) TP, (b) ML, (c) SV, (d) PR. (C) Inter‐group comparison of five taste characteristics: (e) AHS, (f) CTS, (g) NMS, (h) ANS, (i) SCS. *p < 0.05, **p < 0.01, ***p < 0.001, ****p < 0.0001.

To explore the objective taste properties of 4 EFs, *M. lasiocarpa* (ML), *P. rubra* (PR), *S. volubilis* (SV) and *T. palmata* (TP), e‐tongue analysis was conducted on fresh samples, and comparative analyses were performed both among different taste attributes within the same flower (intra‐group) and for the same taste attribute among different flowers (inter‐group) (Figure 2). The *F*‐values, degrees of freedom, and *p*‐values can be found in Table S1.

The intra‐group results showed that CTS and NMS were the most prominent taste characteristics in all four flowers, with values significantly higher than those of the other three indicators in each species (*p* < 0.0001). Nevertheless, each flower exhibited a distinct taste signature. For example, the highest CTS value was observed in *M. lasiocarpa*, whereas *S. volubilis* showed the highest NMS value.

Inter‐group analysis indicated that AHS and SCS remained at low levels across all four flowers and did not differ significantly among species (*p* > 0.05), suggesting that these indices were not effective for distinguishing interspecific taste characteristics. In contrast, CTS, NMS, and ANS exhibited significant inter‐group differences (*p* < 0.05).

#### 3.2.2 Intra‐Group and Inter‐Group Differences in Taste Profiles of Four EFs After Blanching

**FIGURE 3:**
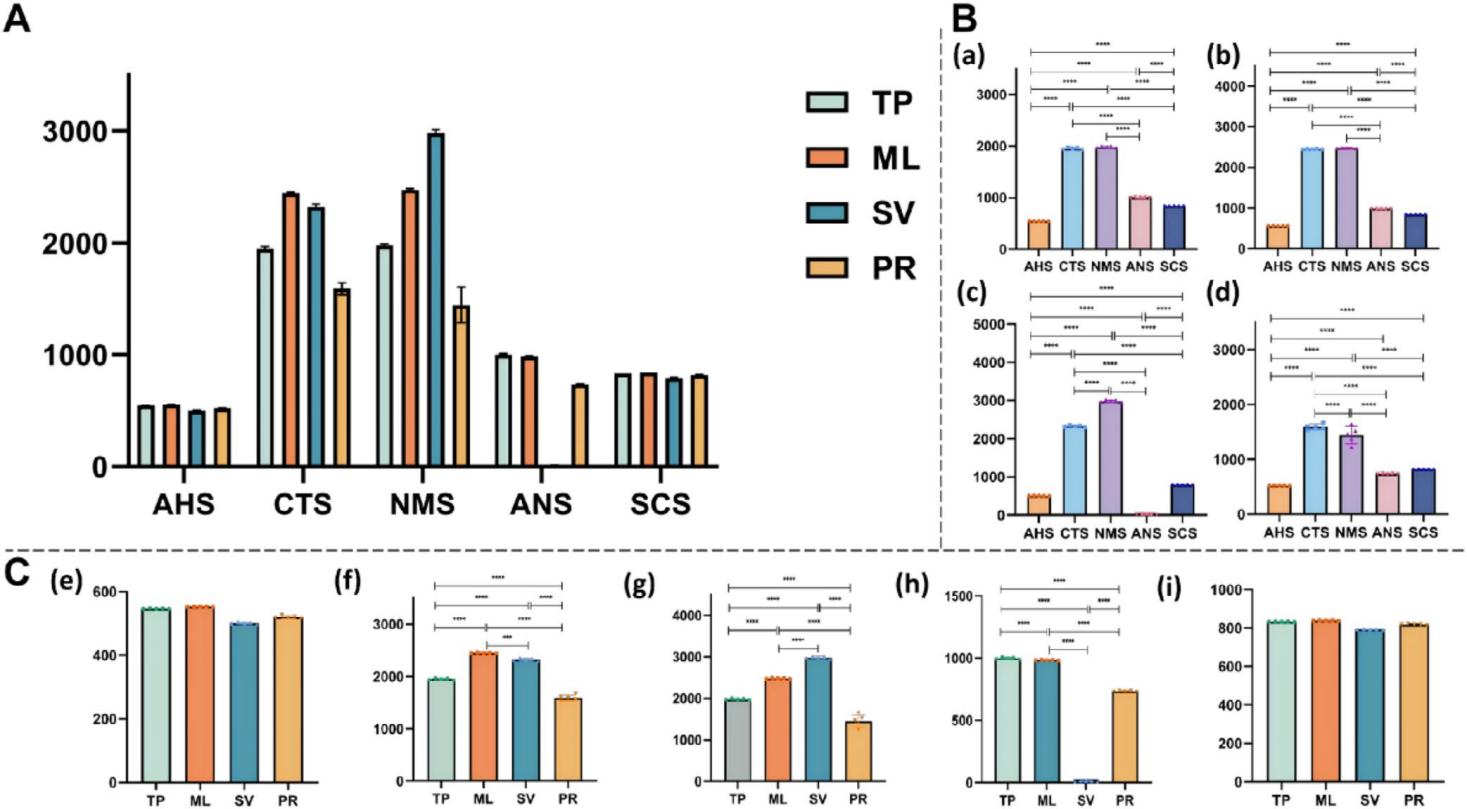
Intra‐group and inter‐group comparisons of four EFs in the blanched group. (A) Overview. (B) Intra‐group comparison of taste attributes: (a) TP, (b) ML, (c) SV, (d) PR. (C) Inter‐group comparison of five taste characteristics: (e) AHS, (f) CTS, (g) NMS, (h) ANS, (i) SCS. ***p < 0.001, ****p < 0.0001.

As the four EFs are commonly blanched prior to consumption by local communities, or consumed after boiling in water for soup or soaking in hot water as a tea substitute, e‐tongue analysis was also conducted on blanched samples (Figure 3). Comparative analyses were performed to examine differences among taste attributes within the same flower (intra‐group) and differences in the same taste attribute among different flowers (inter‐group). The *F*‐values, degrees of freedom, and *p*‐values can be found in Table S2.

Intra‐group analysis showed that CTS and NMS were the most prominent taste characteristics of all four flowers, with values significantly higher than those of the other three indicators across all species (*p* < 0.0001). Inter‐group analysis further indicated that AHS and SCS remained at low levels in all four flowers and did not differ significantly among species (*p* > 0.05), suggesting that these indices were not effective for distinguishing interspecific taste characteristics. In contrast, CTS, NMS, and ANS exhibited significant inter‐group differences (*p* < 0.05).

Overall, blanching treatment significantly modified the overall taste profiles of the four flowers, although each species still retained its characteristic sensory identity after processing.

#### 3.2.3 Comparative Analysis of Taste Profiles of Four EFs Before and After Blanching

**FIGURE 4:**
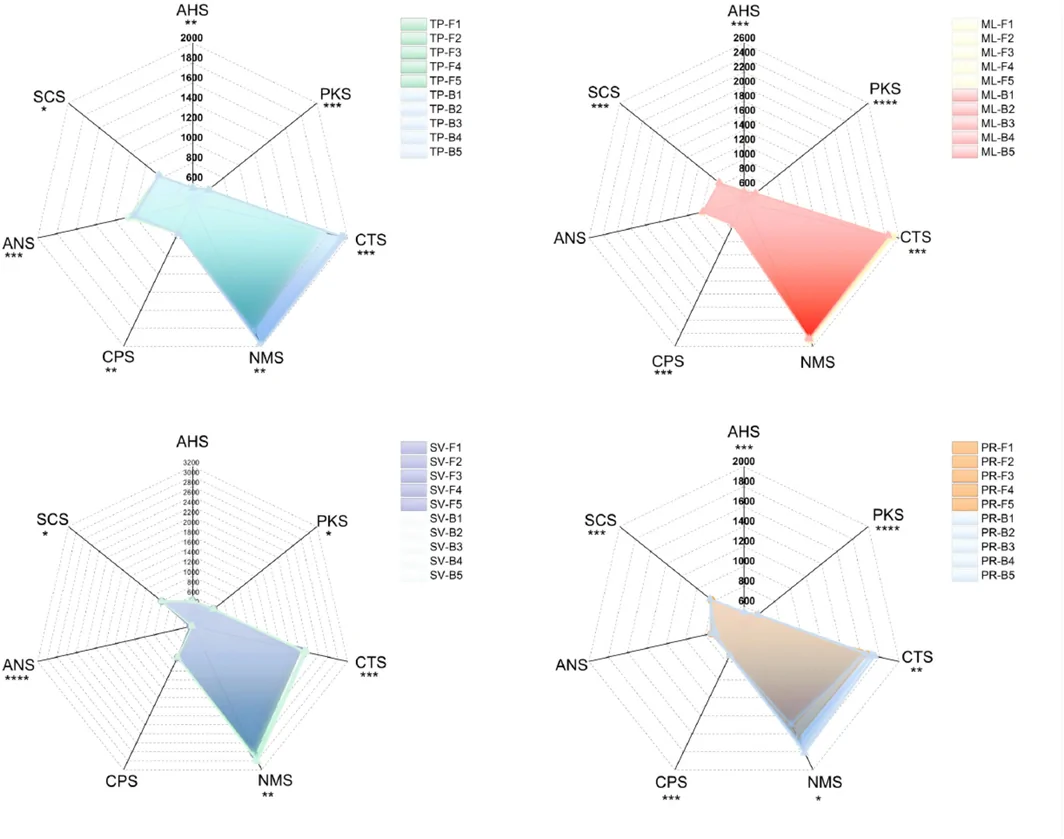
Radar chart comparison of taste components in fresh and blanched samples of four EFs. (A) TP; (B) ML; (C) SV; (D) PR. B, blanched samples; F, fresh samples. *p < 0.05, **p < 0.01, ***p < 0.001, and ****p < 0.0001.

**FIGURE 5:**
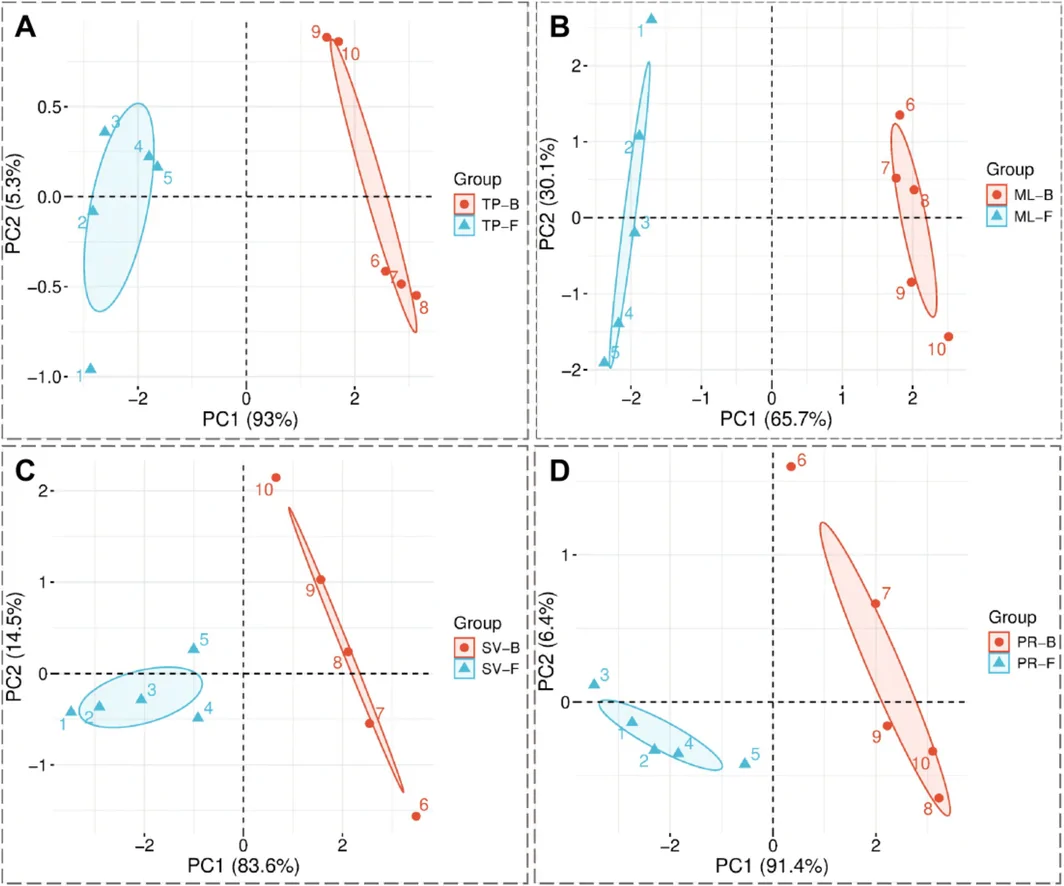
PCA loading plot of taste components in fresh and blanched samples of the four EFs. (A) TP, (B) ML, (C) SV, and (D) PR (B, blanched samples; F, fresh samples).

To more intuitively illustrate the effects of blanching on the overall taste profiles, radar plots were used to visualize the experimental results (Figure 4), and principal component analysis (PCA) was applied to cluster samples and extract key features from the multidimensional taste data (Gao et al. 2022) (Figure 5). It was shown that the overall contours of the radar plots were roughly similar between the fresh samples (Group F) and the blanched samples (Group B). However, there were differences in the taste component level between the fresh and blanched samples of each species, and most of these differences were statistically significant (*p* < 0.05, *p* < 0.01, *p* < 0.001), indicating that blanching treatment could alter the taste component characteristics of the flowers to a certain extent.

Compared with the blanched samples, the fresh samples of TP exhibited higher level of ANS (*p* < 0.001), SCS (*p* < 0.05) and AHS (*p* < 0.01); after blanching, the level of NMS (*p* < 0.01) and CTS (*p* < 0.001) increased significantly. The levels of ANS (*p* < 0.0001) and SCS (*p* < 0.05) in the fresh samples of SV were significantly higher than those in the blanched samples; after blanching, NMS (*p* < 0.01) and CTS (*p* < 0.001) increased significantly. In the fresh samples of PR, the level of AHS and SCS is higher than blanched samples (*p* < 0.001); after blanching, the change trend of its taste components was consistent with that of TP and SV, with the level of NMS (*p* < 0.01) and CTS (*p* < 0.001) increasing significantly. In contrast to the other three flowers, the taste components of ML responded differently to blanching treatment. The CTS signal in its fresh samples was significantly higher (*p* < 0.001); after blanching, the level of SCS and AHS increased significantly (*p* < 0.001), which was opposite to the taste change trend of the other three flowers.

To investigate the effects of blanching on the taste component characteristics of four EFs, PCA was performed on the fresh sample group (Group F) and blanched sample group (Group B) of each species, respectively (Figure 5). The results showed a complete separation between the blanched and fresh samples, with good clustering within each group. This indicates that blanching significantly and consistently altered the overall taste profile, and that the effects were highly reproducible within groups.

There were differences in the principal components driving the variation differed among groups: the TP, PR, and SV groups were all dominated by PC1 as the primary dimension separating the groups, which explained 93%, 91.4% and 83.6% of the total variation, respectively. TP was characterized by decreases in PKS, AHS, and ANS accompanied by increases in CTS and NMS; PR showed increases in AHS, CTS, and CPS with a concomitant decrease in ANS; and SV exhibited decreases in PKS, SCS, and CPS together with increases in NMS and CTS. In contrast, for ML, the separation was jointly driven by two dimensions, PC1 (65.7%) and PC2 (30.1%). PC1 mainly reflected increases in AHS, CPS, and SCS with a decrease in CTS, while PC2 represented a synergistic change of decreased ANS and increased NMS.

The PCA results indicated that blanching could systematically alter the overall taste profiles of the four EFs, and such alterations showed high consistency within each group, suggesting that the regulatory effect of blanching on the taste of EFs was relatively stable. Meanwhile, after blanching, TP, PR and SV all exhibited a similar response pattern dominated by a single principal component (PC1), which was mainly characterized by the synergistic increase in CTS and NMS; whereas ML showed a unique pattern jointly driven by two principal components (PC1 and PC2), with the characteristics of decreased CTS and significant increase in SCS and AHS. This comparison not only verified the specific change of ML observed in the radar plot analysis but also confirmed from the perspective of multivariate analysis that although blanching could generally regulate the intensity of taste components, it simultaneously highlighted and preserved the fundamental differences in taste characteristics among various species.

## 4 Discussion

### 4.1 Edible Flowers as Food but More Than Food

The consumption of edible flowers has emerged as a fashion dietary culture in China with Yunnan Province as the core zone. Yunnan is renowned for its biological and cultural diversity. The majority of raw materials and products of EFs are sourced from this province, together with their traditional culture (Zhang, Zhang, et al. 2025). However, it is important to note that many EFs are not particularly appetizing. The four species selected for this study, namely *Musella lasiocarpa*, *Plumeria rubra* , *Stephanotis volubilis* and *Trevesia palmata*, were found to be lacking in gustatory appeal, as evidenced by both the villagers' verbal accounts (Table 1) and the results of e‐tongue tests. Even *S. volubilis* and *T. palmata* taste bitter and not acceptable to many people.

It has been proven that taste significantly influences food selection and acceptance, while the perception of taste is shaped by factors such as culture and life experiences (Hossain et al. 2025). The apparent paradox relationship that flowers with limited palatability persist as valued dietary components may suggest that the cultural significance of flower‐eating can transcend their sensory limitations. In Yunnan's food culture, “edibility” is defined by a complex interplay of tradition, seasonal rhythm, and symbolic meanings. These flowers are consumed not because they taste good by general standards, but because they taste culturally meaningful (Mintz 1986). Their flavors, even when unpalatable, are markers of identity of Yunnanese, seasonality perception and connection, as well as continuation of flower‐eating customs (Rozin and Vollmecke 1986). In this sense, these flowers are treated as food rich in nutrients but more than food.

### 4.2 Nutritional Potential of Four Edible Flowers as Overlooked Plant Food Organs

Conventional classifications of plant foods typically list roots (including tubers and rhizomes), leaves, stems and shoots, fleshy fruits, grains, seeds, and nuts, while floral organs are frequently omitted (Turner et al. 2011). Nutritional studies on the floral organs of diverse plant species from different regions have shown that, similar to the leaves and seeds of edible plants, flowers also possess a well‐balanced macronutrient composition, particularly among indigenous species (Sotelo et al. 2007). In this study, the four EFs exhibited a distinct nutritional profile characterized by particularly high dietary fiber and moderate mineral richness, whereas their protein, energy, fat, and carbohydrate levels were lower than those of broccoli after conversion to the same dry‐weight basis. Nevertheless, their energy and protein content remained higher than those reported for several globally studied EFs such as dahlia, rose, calendula and centaurea (Pires et al. 2017). However, with the exception of dietary fiber, most of these macronutrient indicators were still lower than those reported for certain wild medicinal food plants traditionally consumed in Yunnan, such as *Disporopsis aspersa* and *Maianthemum atropurpureum* (Chen et al. 2025; Xu et al. 2021). These nutritional attributes, particularly the high fiber and essential mineral contents, suggest that these EFs hold promise as functional food ingredients or nutraceutical sources for managing chronic diseases such as obesity, diabetes, and cardiovascular disorders (Reynolds et al. 2026). Although carbohydrates have been reported as the most abundant macronutrient in some nutritional analyses of EFs (Pires et al. 2017; Sommano et al. 2025), this pattern was not observed in this study. In all four species, carbohydrate contents were lower than dietary fiber, and in *S. volubilis* and *T. palmata* they were even lower than protein contents, a pattern consistent with that reported for *M. atropurpureum* . There is higher proportion of carbohydrates in these two EFs from Thailand (Sommano et al. 2025), suggesting potential regional variation linked to ecological context.

All four EFs contained trace elements except selenium (Se). It aligns with the trace element profiles reported for many other EF species, supporting the view that EFs constitute a valuable dietary source of trace elements (Drava et al. 2020; Rop et al. 2012). Previous studies have shown associations between mineral element content and bioactive compounds; for example, EFs with higher Cu levels often exhibit higher total flavonoid content, and antioxidant activity has been attributed in part to minerals such as Cu, Fe, and Mg (Mlcek et al. 2021). Building on the quantified mineral element data obtained in this study, further investigation of the links between nutritional composition and antioxidant capacity may provide clues to the potential roles of EFs in supporting human metabolic health.

Vitamin A is a fat‐soluble vitamin essential for maintaining normal metabolism and physiological functions in the human body. Some studies have suggested that yellow flowers are generally rich in vitamin A (Mlcek and Rop 2011). However, despite the yellow coloration of the inflorescences and bracts of *M. lasiocarpa* and the corollas of *P. rubra* examined in this study, vitamin A was not detected in these samples. Vitamin C, an important growth factor and antioxidant that promotes iron absorption, strengthens blood vessels, and accelerates wound healing (Rachkeeree et al. 2018), was relatively abundant in *S. volubilis* . Vitamin E is also an effective antioxidant in the human body, and it was consistently detected in all four EFs in this study, suggesting their potential antioxidant capacity.

The amino acid profiles also support the nutritional significance of these flowers. All four EFs meet the WHO/FAO ideal amino acid standard, indicating that they can be regarded as high‐quality and nutritionally favorable sources of dietary amino acids. The E/T ratio of *P. rubra* is comparable to that of medicinal food plants *D. aspersa* and *M. atropurpureum* , while the other three species exhibit even higher ratios (Chen et al. 2025; Xu et al. 2021). Additionally, they exhibit the highest concentration of lysine (Lys), followed by aspartic acid (Asp) and glutamic acid (Glu). Lys plays an important role in promoting human growth and development, enhancing immune function, and facilitating the absorption of certain nutrients (Liang et al. 2023). However, it is present in very low amounts in cereal proteins and is the limiting amino acid in all the main cereal species (Leinonen et al. 2019). The relatively high Lys contents detected in *S. volubilis* , *T. palmata*, and *M. lasiocarpa* suggest that consuming these EFs in combination with staple grains is nutritionally and scientifically sound. This finding leads to support for the nutritional rationale underlying the long‐standing dietary practice in Yunnan of treating flowers as vegetables and integrating them into daily diet.

### 4.3 Taste and Choice of Edible Flowers

The e‐tongue results can be interpreted within established theories of human taste perception, particularly those concerning amino acid‐derived umami sensation and the interaction of umami, saltiness, and bitterness in shaping overall taste experience (Keast and Breslin 2003; Kemp and Beauchamp 1994). E‐tongue analysis indicates that the four EFs share a fundamental taste profile characterized by high CTS (saltiness) and NMS (umami) values. This profile aligns with their generally rich content of flavor‐active amino acids (Biesalski 2026; Gary 2015). For example, aspartic acid and lysine are present in higher concentrations, which may partly account for their common umami flavor characteristics (He et al. 2024). Taste profoundly influences human food choice (Breslin 2013). The umami‐salty base likely serves as an important flavor foundation, potentially contributing to their acceptance as routine food ingredients.

*S. volubilis* exhibited the highest NMS value among the four EFs, along with a relatively high CTS value, while its SCS value was moderate and its ANS value was extremely low. This pattern is of interest as local descriptions identify bitterness as the dominant taste of *S. volubilis* , with little to no sweetness perceived. Accordingly, the low ANS values observed in this study were consistent with expectations. The fact that SCS values were lower than both NMS and CTS values may be associated with the higher hydrophobicity of bitter compounds, which limits their solubility in the aqueous solutions used for e‐tongue analysis (Kobayashi et al. 2010).

Moreover, interactions among different taste modalities have been confirmed through human sensory evaluation as well as neural recordings from the chorda tympani (CT) and the parabrachial nucleus (PbN) (Adachi and Aoyama 1991; Formaker et al. 2004; Kim et al. 2015). A phenomenon termed “mixture suppression,” which refers to the reduction in perceived intensity when two or more suprathreshold taste stimuli are combined, resulting in an overall intensity lower than the sum of individual intensities (Keast and Breslin 2003). Previous studies further indicate that umami interacts with other tastes, with one notable characteristic being its ability to suppress bitterness (Beauchamp 2024; Kemp and Beauchamp 1994; Noguchi et al. 1975). This interaction may provide another important explanation for why the SCS values detected by the e‐tongue were lower than expected, as umami and saltiness could suppress the “expression of bitterness.” Such suppression is particularly relevant because bitterness generally exerts a negative influence on food choice and intake in humans (Breslin 2013).

These interactions may also help explain why local communities continue to favor the consumption of these bitter flowers. Given that humans are especially sensitive to bitterness (Han et al. 2018), the perception of umami may be partially masked by bitterness during actual consumption. However, without the strong underlying umami and salty taste components, these flowers might not be chosen as foods at all and would perhaps be regarded solely as medicinal plants (Silva et al. 2024). Similarly, *T. palmata* is locally described as intensely bitter upon first taste, yet its NMS and CTS values were also markedly higher than its SCS value. An integrated interpretation of these complex taste profiles together with local sensory reports therefore provides a plausible explanation for the regional preference for consuming *S. volubilis* and *T. palmata*.

### 4.4 Effect of Blanching on the Taste of Edible Flowers

The radar plots and PCA results confirmed that, although blanching significantly altered the intensity of certain taste components, it did not change the overall profile of taste characteristics or the pattern of inter‐group differences among the four flowers. Notably, *M. lasiocarpa* responded to blanching in a fundamentally different way compared with the other three flowers, indicating that processing effects do not uniform across EFs. The retention of these sensory characteristics may help explain why these flowers have been consistently accepted and utilized in local diets over time.

In this context, local blanching practices are likely to function primarily as a habitual preprocessing step rather than as a deliberate method for fine‐tuning flavor. Similar to the blanching of spinach to reduce oxalic acid and soften texture (Chai and Liebman 2005), blanching may serve to dissolve or hydrolyze potential irritants or toxic components, inactivate enzymes, and extend the edible period, with changes in flavor emerging as secondary and largely unintended outcomes rather than the primary objective (Fante and Noreña 2012; Gonçalves et al. 2010; Wang et al. 2020).

The changes in taste induced by blanching inevitably involve transformations in underlying chemical components. Such transformations are not only reflected in the intensity of taste signals detected by the electronic tongue, but also imply that during thermal processing, the decomposition, transformation, or interaction of complex components in plant tissues, such as volatile flavor compounds, taste‐active amino acids, organic acids, and even polyphenols (Ijod et al. 2025; Shatsala Wangusi et al. 2025; Nchangwe et al. 2024). In subsequent studies, techniques such as GC–MS (Tian et al. 2026; Wang et al. 2025) and LC–MS (Hughey et al. 2012; Kim et al. 2025; Li, Xie, et al. 2025) could be employed to investigate changes in chemical composition before and after blanching, elucidating the variation patterns of key flavor components and toxic substances during blanching and other processing methods. Such research would provide a scientific basis for the sustainable use of local EF resources and help to clarify the empirical understanding embedded in local knowledge systems regarding plant processing and consumption.

### 4.5 Limitations and Prospects of Edible Flowers Studies

The nutritional significance of these EFs should be considered beyond compositional data alone. Although the four species were characterized by high dietary fiber contents and appreciable levels of some minerals, the physiological benefits of these nutrients ultimately depend on their bioaccessibility and bioavailability. Plant‐based foods commonly contain anti‐nutritional factors such as oxalates, phytates, and tannins, which can form insoluble complexes with minerals including Ca, Cu, and Mn, thereby reducing their net absorption in the gastrointestinal tract (Samtiya et al. 2020). The common practice of blanching or boiling may reduce water‐soluble nutrients such as vitamin C through leaching, while fat‐soluble vitamins including vitamin E may be less vulnerable (Lee et al. 2018). Nevertheless, the effects of culinary processing on nutrient retention are highly dependent on the specific nutrient, vegetable matrix, and cooking method, and the impact on bioavailability in EFs remains largely unknown.

Moreover, field observations indicate that EFs in Yunnan are generally consumed seasonally, in relatively small quantities, and as part of a diverse repertoire of plant foods rather than as staple foods. Their contribution to local diets may therefore derive not only from the provision of specific nutrients but also from their role in enhancing dietary diversity and broadening the range of plant resources available for consumption. Evaluating their nutritional contribution under realistic dietary conditions therefore represents another important direction for future research.

While the e‐tongue provides objective and reproducible taste profiles, it does not replace human sensory perception, particularly the hedonic and cultural dimensions that shape food acceptance. A limitation of this study is the absence of human sensory validation (e.g., trained panels or consumer tests), and such validation would be essential to confirm how these taste profiles are actually perceived and valued by local communities and potential consumers. Nevertheless, the e‐tongue successfully revealed a hidden taste foundation, namely umami and saltiness, which appears to underlie the local preference for these bitter‐tasting flowers, suggesting that their consumption is not merely a matter of cultural habit but also has a sensory basis. These findings may thus serve as scientific support for promoting these flowers as regional specialty foods. However, future studies should integrate instrumental analysis with trained sensory panels or consumer tests to provide complementary evidence and to guide product development and marketing strategies more effectively.

Beyond these methodological considerations, future EF research may also benefit from a clearer focus on the fundamental concept of edibility. The nutritional profiles documented in this study do suggest that these traditionally consumed EFs possess characteristics relevant to functional food development. However, as many studies emphasize bioactive compounds, antioxidant properties, and metabolomic profiles, EFs have become a promising plant resource for functional foods and natural remedies (Rao and Poonia 2024; Breda et al. 2025; Marchioni et al. 2024; Rinaldi et al. 2025). The original meaning of the term “edible flowers,” that is, flowers that are safe and appropriate for human consumption, risks being diluted by expectations of EFs development and commercial success (Oberholzer et al. 2025). Our research returns to the original, food‐oriented foundation of edibility by focusing explicitly on four flower species that are traditionally consumed as food in Yunnan, as documented through prior ethnobotanical investigation (Zhang, Cheng, et al. 2023). By integrating nutritional composition analysis with e‐tongue assessment, we evaluated these flowers as foods, emphasizing their nutritional and sensory characteristics rather than their potential as sources of isolated functional compounds. Such an approach highlights the importance of examining EFs within the broader contexts of dietary practices, local knowledge, and food systems, thereby contributing to a more comprehensive understanding of their roles in biocultural food traditions.

## 5 Conclusions

This study provides an integrated evaluation of traditionally consumed edible flowers of four species (*Musella lasiocarpa*, *Plumeria rubra* , *Stephanotis volubilis*, and *Trevesia palmata*) from Yunnan by combining nutritional composition analysis with e‐tongue taste profiling. The results demonstrate that all four flowers possess noteworthy nutritional value, with high levels of dietary fiber, favorable amino acid profiles, and appreciable levels of minerals and vitamins (K, Ca, Mn, Cu, and vitamin E). Compared with broccoli, a widely recognized nutritious vegetable, these EFs exhibited higher dietary fiber contents and comparable or higher concentrations of several mineral elements on a dry‐weight basis, supporting their role as nutritionally meaningful plant foods rather than underutilized flowers from marginalized communities. Electronic tongue analysis showed that the four EFs share a similar umami–salty taste foundation. However, in *S. volubilis* and *T. palmata*, this underlying flavor may be masked by pronounced bitterness during consumption, while the subtle presence of umami and saltiness may nonetheless be one of the key reasons these bitter flowers have been persistently favored and widely used in local food practices. E‐tongue further revealed that the four EFs display distinct and stable taste characteristics. Although blanching significantly altered the intensity of certain taste components, it did not change the overall taste contours or the inter‐species differentiation patterns. These findings indicate that common local processing practices modify, but do not homogenize, the sensory profiles of EFs.

The combination of nutritional and flavor characteristics provides a material basis for the long‐term selection and consumption of these flowers in local food systems. Given the poor taste, cultural factors are more likely to be the driving force behind the continued consumption of flowers by local people. By situating nutritional and sensory data within traditional processing practices, this study offers a scientifically grounded interpretation of local knowledge embedded in flower‐eating traditions. In Yunnan and many places worldwide, EFs represent a form of biocultural diversity in which species diversity, local knowledge, and cultural practices are mutually constitutive (Khomdram et al. 2019; Manzanero‐Medina et al. 2026; Motti et al. 2022; Mulík and Ozuna 2020). This perspective foregrounds that EFs are not merely a type of utilized plant resources but nodes in local food systems that contribute to human nutrition, dietary diversity, food security and cultural identity. By focusing on the fundamental attributes of edibility, including nutritional value, taste characteristics, and cultural embeddedness, this study contributes to a more contextually grounded understanding of EFs and provides a framework for future research and the sustainable utilization of these unique food resources.

## Author Contributions

**Yan Li:** methodology, data curation, writing – original draft, validation, formal analysis, visualization. **Zhuo Cheng:** visualization, software. **Chunlin Long:** writing – review and editing, supervision, funding acquisition, conceptualization. **Sarana Rose Sommano:** funding acquisition, writing – review and editing. **Miaomiao Wang:** data curation, formal analysis, methodology. **Qing Zhang:** writing – original draft, project administration, writing – review and editing, formal analysis. **Zhengjiang Xian:** investigation, resources. **Yingzai Zhou:** investigation, resources. **Wenqi Wang:** methodology, data curation.

## Funding

This research was funded by the Yunnan Provincial Sci & Tech Talent and Platform Program (202305AF150121), the National Natural Science Foundation of China (32370407), the National Social Science Foundation of China (25&ZD168), the Mekong‐Lancang Cooperation Program (HX2023052) under the project “Sustainable conservation and value adding locally edible floral species in the Mekong regions,” the Graduate Research Projects of Minzu University of China (BZZKY‐X2025016), the Chinese Government Scholarship (CSC 202606390029) and the Strategic Research and Consultancy Project of the Chinese Academy of Engineering (2025‐PP‐12).

## Ethics Statement

The authors have nothing to report.

## Conflicts of Interest

The authors declare no conflicts of interest.

## Supporting information


**Table S1:** Two‐way ANOVA results for taste characteristics of fresh EFs.
**Table S2:** Two‐way ANOVA results for taste characteristics of blanched EFs.

## Data Availability

All data generated or analyzed during this study was included in this published article.
